# Matrix Metalloproteinases are required for membrane motility and lumenogenesis during *Drosophila* heart development

**DOI:** 10.1371/journal.pone.0171905

**Published:** 2017-02-13

**Authors:** Qanber S. Raza, Jessica L. Vanderploeg, J. Roger Jacobs

**Affiliations:** 1 Department of Cell Biology, School of Medicine, University of Pittsburgh, Pittsburgh, Pennsylvania, United States of America; 2 Department of Biology, Taylor University, Upland, Indiana, United States of America; 3 Department of Biology, McMaster University, Hamilton, Ontario, Canada; University of Dublin Trinity College, IRELAND

## Abstract

Matrix Metalloproteinases (Mmps) degrade glycoproteins and proteoglycans of the extracellular matrix (ECM) or cell surface and are crucial for morphogenesis. Mmps and their inhibitors are expressed during early stages of cardiac development in vertebrates and expression is altered in multiple congenital cardiomyopathies such as cardia bifida. *Drosophila* genome encodes two copies of Mmps, Mmp1 and Mmp2 whereas in humans up to 25 Mmps have been identified with overlapping functions. We investigated the role of Mmps during embryonic heart development in *Drosophila*, a process which is morphogenetically similar to early heart tube formation in vertebrates. We demonstrate that the two Mmps in *Drosophila* have distinct and overlapping roles in cell motility, cell adhesion and cardiac lumenogenesis. We determined that Mmp1 and Mmp2 promote Leading Edge membrane dynamics of cardioblasts during collective migration. Mmp2 is essential for cardiac lumen formation, and mutants generate a cardia bifida phenotype. Mmp1 is required for luminal expansion. Mmp1 and Mmp2 both localise to the basal domains of cardiac cells, however, occupy non-overlapping domains apically. Mmp1 and Mmp2 regulate the proteoglycan composition and size of the apical and basal ECM, yet only Mmp2 is required to restrict ECM assembly to the lumen. Mmp1 negatively regulates the size of the adhesive Cadherin cell surface domain, whereas in a complementary fashion, Mmp2 negatively regulates the size of the Integrin-ECM domain and thereby prescribes the domain to establish and restrict Slit morphogen signalling. Inhibition of Mmp activity through ectopic expression of Tissue Inhibitor of Metalloproteinase in the ectoderm blocks lumen formation. Therefore, Mmp expression and function identifies ECM differentiation and remodelling as a key element for cell polarisation and organogenesis.

## Introduction

Matrix Metalloproteinases (Mmps) are zinc dependent proteases which digest components of the extracellular matrix (ECM) and embedded signalling molecules. Mmps enable morphogenesis by modifying cell migration, cell polarization, ECM remodelling and lumenogenesis [1]. Mmps are regulated by signalling pathways such as Wnt/β-catenin and can modulate signalling of growth factors such as Vascular Endothelial Growth Factors (VEGF) [2–4]. Due to genetic redundancy in the mammalian genome, it is challenging to examine Mmp function when genetic compensation can affect mutant analysis [1, 5–7]. In contrast, the *Drosophila* genome encodes two Mmps, a secreted protease Mmp1 and a glycophosphatidylinositol (GPI) anchored Mmp2 [8, 9]. *Drosophila* has a single Tissue Inhibitor of Metalloproteinase (Timp), which has been shown experimentally to be a potent inhibitor of vertebrate Mmps, *Drosophila* Mmp1 and Mmp2, and other extracellular proteases [10].

An *in vitro* model of human vasculogenesis reveals that endothelial cells require a membrane associated Mmp (MT1-Mmp) for luminal expansion and formation of vascular guidance tunnels [11]. Branching morphogenesis in organs such as lungs, mammary and submandibular glands requires activity of Mmps for cell motility and lumen formation, mediated by ECM degradation [12]. During tumour invasion, MT1-Mmp, targeted to the invadopodia, promotes metastasis by degrading ECM barriers [13, 14]. Although vertebrate Mmps have received considerable attention, their contributions to embryonic morphogenesis are less characterised because of the barriers to genetic approaches [5, 7, 15, 16]. Using *Drosophila* embryogenesis as a genetic model, we can study the effect of complete elimination or inhibition of Mmp activity *in vivo*. Conservation of activity in *Drosophila* homologues of Mmps has been well established [8, 9]. Intriguingly however, Mmp activity is not required for embryonic viability in *Drosophila* since single or double *mmp* mutant embryos hatch and survive until mid or late larval stages [17]. Nevertheless, developmental processes such as motor axon fasciculation during embryogenesis require Mmp activity [18, 19]. Expression data demonstrated that Mmps are upregulated during late stages of embryogenesis in multiple tissues [17]. Vertebrate Mmps and Timps are expressed in the cardiomyocytes during early heart tube assembly [20, 21] and modulate cardiac morphogenetic events such as heart tube formation, directional looping [22] and differentiation of ostial cells [23].

In this study, we tested the genetic requirement of Mmps during heart development in *Drosophila*, a process with striking similarities with early stages of vertebrate cardiogenesis [24, 25]. To form the embryonic heart, cardioblasts (CBs) in the lateral mesoderm align into bilateral rows and collectively migrate towards the dorsal midline. There, the CBs form specific medial adhesions with their contralateral partners and form an apical lumen, which expands as the embryo transitions through larval stages [26]. We demonstrate that Mmp1 and Mmp2 are expressed in the CBs and both influence Collective Cell Migration (CCM) of CBs by regulating lateral adhesions between CBs and the activity of the migratory Leading Edge (LE). After migration, Mmp1 and Mmp2 play complementary roles during ECM remodelling, cell polarization and lumen formation. Mmp2 is essential for forming adhesions with contralateral CBs. Mmp1 is required to limit the domain of E-Cadherin based contralateral adhesions and its activity contributes to luminal expansion. We show that Mmp2 regulates apical localization of guidance signalling molecules Slit and Robo, and ECM receptors Integrin and Dystroglycan (Dg). Composition of the CB apical luminal ECM is modulated by both Mmps, whereas positioning is only dependent on Mmp2. Overall, we demonstrate that Mmps play essential roles promoting CCM, ECM remodelling, cell polarization and lumen formation during *Drosophila* cardiogenesis.

## Results

### *mmp1* and *mmp2* mutants have distinct heart phenotypes

**Fig 1:**
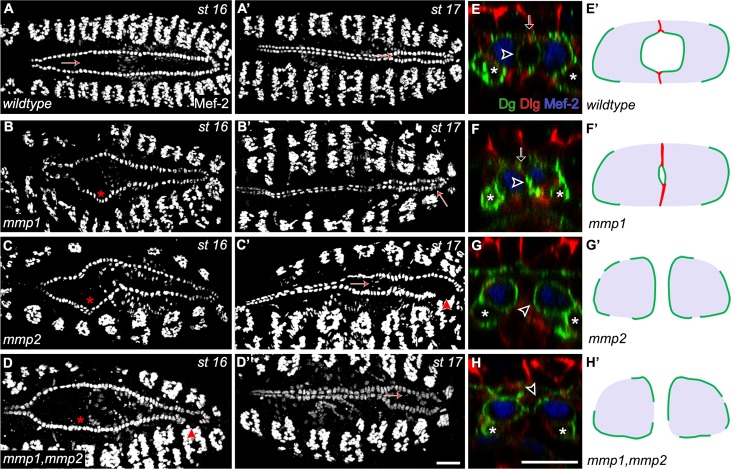
MMPs are required for embryonic heart development and lumen formation. (A) In stage 16 wildtype embryos labelled with MEF-2 antibody, CBs form bilateral rows and migrate collectively towards the dorsal midline (arrow). (A’) At stage 17, contralateral CBs align at the midline and form a lumen (arrow). (B-B’) In *mmp1*^*Q112*^*/ mmp1*^*Q112*^ null mutants (from here on referred to as *mmp1*), organization of CB rows is uneven and delayed migration of CBs is observed (asterisk), however they reach the midline and form a reduced size lumen (arrow). (C-D’) In *mmp2*^*w307*^*/ mmp2*^*w307*^ and *mmp1*^*q112*^, *mmp2*^*w307*^*/ mmp1*^*q112*^, *mmp2*^*w307*^ null mutants (from here on referred to as *mmp2* and *mmp1*,*mmp2*, respectively), organization of CBs along the bilateral rows is disrupted and CB migration is delayed (asterisk). The CBs in the heart region do not reach the midline by stage 17 (arrow). (E-H) Cross sectional views of stage 17 hearts labelled with α-Mef-2, α-Dg and α-Dlg are visualised (E-H). (E) In wildtype, Dg localises to the CB luminal domain (arrowhead) and pericardial cells (asterisk) while Dlg localises to the junctional domains (arrow). (F) In *mmp1* mutants, a reduced Dg labeled lumen forms (arrowhead) and Dlg labels the enlarged junctional domain (arrow). (G) In *mmp2* and *mmp1*,*mmp2* mutants, CBs fail to adhere to contralateral partners (arrowhead). CBs exhibit rounded morphology and Dg localises along an extended apical domain (arrowhead). (E’-H’) Representation of CBs at stage 17 in wildtype and *mmp1*, *mmp2* and *mmp1*,*mmp2* mutants summarising distribution of Dg (green), Dlg (red) and Mef2 (blue). Posterior is to the right in dorsal view images (A-D). Scales—25 μm in A-D’ and 10 μm in E-H’.

In *Drosophila*, polarised positioning of molecularly distinct ECMs to the basal and luminal domains is essential for the formation of the heart [27–29]. Since Mmps contribute to ECM degradation and remodeling in other systems, we reasoned that Mmps may contribute to *Drosophila* heart formation. To address this hypothesis, we examined the phenotypes of loss of function single and double *mmp1* and *mmp2* mutant hearts with luminal, junctional and nuclear markers and assessed embryonic heart structure (Fig 1). The cardioblasts of stage 16 wildtype embryos were aligned in bilateral rows and migrated to the midline collectively (Fig 1A). At stage 17, contralateral CBs contact at the midline and then reshape to enclose a medial lumen (Fig 1A’). Dystroglycan (Dg), an ECM receptor, localises to the luminal domain, whereas Discs-large (Dlg), an apical polarity scaffold protein, labels the junctions at the apical attachment sites (Fig 1E and 1E’). Although loss of either or both *mmp1* and *mmp2* resulted in disorganised CB arrangement along the bilateral rows, CBs eventually reached the midline (Fig 1B, 1B’,1C, 1C’, 1D and 1D’). Following migration, *mmp1* and *mmp2* mutant phenotypes diverge. In *mmp1* mutants, a Dg rich lumen forms, but it is reduced in size and is enclosed by extended Dlg marked cell junctions (Fig 1F and 1F’). In *mmp2* and *mmp1*,*mmp2* double mutants, a lumen fails to form (Fig 1G, 1G’, 1H and 1H’). Prior to lumen closure, wildtype CBs curve to form a medial pocket. CB somas in *mmp2* mutants are spherical and Dg localisation is circumferentially distributed, whereas Dlg marked junctions are absent (Fig 1G, 1G’, 1H and 1H’). We scored 20–25 βPS Integrin labelled posterior heart cross-sections and determined that 75% of the embryos mutant for either *mmp2* or both *mmp1*,*mmp2* were rounded and lacked contralateral contacts, whereas the most prevalent phenotype in *mmp1* mutants was the presence of a small apical lumen (Table 1). These results suggest that Mmp2 is required to develop a heart tube, whereas Mmp1 is required for luminal expansion.

**Table 1:** Lumen formation defects in *mmp* mutants.

*Genotypes*	Normal Lumen (%)	Reduced Lumen* (%)	No Lumen † (%)	Rounded CBs ‡ (%)	Embryos Scored (n)
*wildtype*	95	5	0	0	21
*mmp1*	16	68	8	8	25
*mmp2*	0	9	14	77	22
*mmp1*,*mmp2*	5	15	5	75	20

*—Localises luminal markers, but the lumen width is less than 2 μm

† - The apical medial region does not localise luminal markers

‡ - Contralateral CBs do not make contact

### Remodelling of apical and basal ECM requires Mmp1 and Mmp2 activity in the CBs

**Fig 2:**
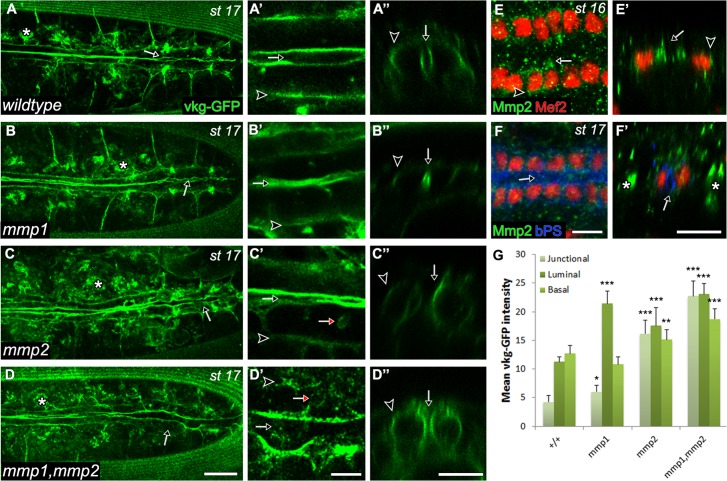
MMP2 is required to define the boundaries of Collagen IV deposition. (A-D) Dorsal, (A’-D’) enlarged and (A”-D”) cross sectional views of live *vkg-GFP* embryos at stage 17 are shown. (A-A”) In wildtype embryos, Vkg (Collagen IV) localises to the luminal (arrow) and basal domains (A’ arrowhead). (B-B”) In *mmp1* mutants, Vkg localises to the reduced luminal (arrow) and basal domains (arrowhead). (C”,D”) In *mmp2* and *mmp1*,*mmp2* mutants, Vkg localises to the entire apical domain (arrow) and basal domains (arrowhead). Ectopic lumens between lateral domains of CBs are observed in *mmp2* and *mmp1*,*mmp2* mutants (B’-D’ red arrows). (E,F) Dorsal and (E’,F’) cross sectional views of the heart in wildtype. (E-E’) Mmp2-GFP localises to the leading edge (arrows) and basal domain (arrowheads) of CBs during migratory stages. (F-F’) Mmp2 does not localise to the βPS integrin labeled luminal, basal domains or junctional domains of the CBs at stage 17 (arrows). (G) Mean Vkg-GFP fluorescence intensity in wildtype and *mmp* mutants are shown for junctional, luminal and basal domains. Asterisks indicate significant differences in fluorescence intensity between wildtype and mutants for each domain, respectively, as determined by one-tailed T test. *—p<0.05, **—p<0.01, ***—p<0.001. (A-D, F’) Embryonic hemocytes are marked with asterisks. Scales—25 μm in A-D, 10 μm in A’-D” and E-F’.

In *Drosophila*, Mmp2 is required to cleave components of the basement membrane and regulate its polarised deposition [19, 30–33]. To explore Collagen-IV deposition in the developing heart, we analysed time-lapse movies of embryos expressing a CollagenIV-GFP gene trap construct (*VkgGFP*). In wildtype embryos, Vkg is present at the basal and medially oriented apical surface of the CBs (Fig 2A, S1 Movie). At the apical side of CBs, Vkg is absent from the apical extensions and is restricted to the pre-luminal domain during CCM, and to the lumen thereafter (Fig 2A”). In *mmp1* mutants, VkgGFP localises normally and is absent from the adhesive domains (Fig 2B–2B”, S2 Movie). In *mmp2* and *mmp1*,*mmp2* double mutants, VkgGFP localisation extended to the entire apical region of the CBs (Fig 2C–2C” and 2D–2D”, S3 and S4 Movies). The junctional (the dorsal-apical domain of CBs which adheres to contralateral CBs), luminal (the apical domain encompassing the lumen), basal and lateral (domain connecting ipsilateral CBs) domains in *mmp2* and *mmp1*,*mmp2* mutants significantly accumulated GFP signal relative to wildtype (Fig 2C’,2D’ and 2G). We reasoned that if spatial restriction of ECM around the CBs requires Mmp2, then Mmp2 would not be active in the pre-luminal domain. By employing a Mmp2-GFP construct [31] we determined that Mmp2 was enriched in the apical outgrowths and basal domains and was absent from the pre-luminal domain (Fig 2E and 2E’). After heart assembly, enriched localisation of Mmp2 was not detected at any particular CB cell domain (Fig 2F and 2F’). Mmp1 was detected in the pre-luminal and luminal domain of CBs during migratory and lumen formation stages, respectively, supporting a model in which Mmp1 excludes Cadherin from this domain (S1 Fig).

**Fig 3:**
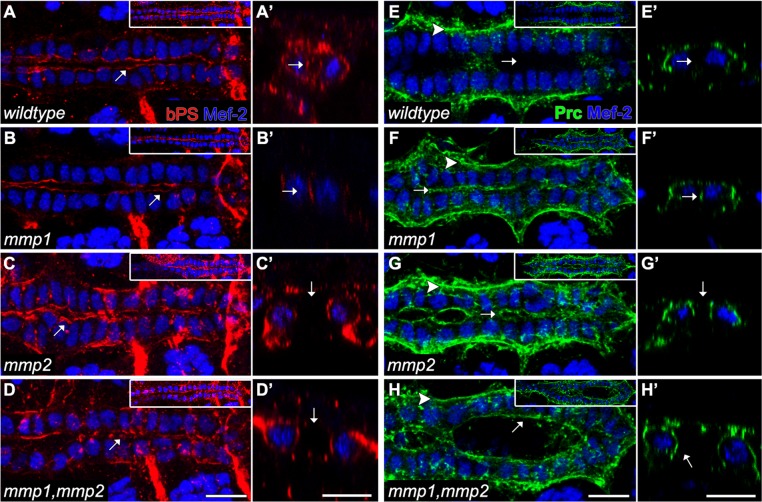
Apical and basal markers are mislocalised in *mmp* mutants. Wildtype and *mmp* mutant embryos labelled with α-βPS(A-D’, red), α-Prc (E-H’,green) and α-Mef2 (A-H’, blue) are shown from both (A-H) dorsal and (A’-H’) cross sectional views of the heart. (A-A’) In wildtype, βPS localises to the luminal domain (arrow). (B-B’) In *mmp1* mutant embryos, βPS is localised to the reduced lumen (arrow). (C-D’) In *mmp2* and *mmp1*,*mmp2* mutant embryos, βPS covers the entire medial apical surface of the CBs (arrow). (E-E’) In wildtype, Pericardin localises exclusively to the basal domain (arrowhead). In *mmp1* (F-F’), *mmp2* (G-G”) and *mmp1*,*mmp2* (H-H’) mutants, Prc localises to the entire medial surface (arrow) as well as the basal side (arrowhead) of the CBs. Scales—10 μm.

If Mmp2 limits the extent of the luminal ECM, does it also play a role in defining ECM identity? To address this question we examined the distribution of Pericardin (Prc), an ECM component expressed and deposited by the pericardial cells exclusively at the basal side of CBs [29]. In contrast to wildtype, where Prc is excluded from the lumen (Fig 3E and 3E’), we found Prc localisation at the apical ECM as well as the basal ECM in *mmp1*, *mmp2*, and *mmp1*,*mmp2* mutants (Fig 3F, 3F’, 3G, 3G’, 3H and 3H’). These results suggest that Mmp1 and Mmp2 collectively regulate the identity of the apical ECM, whereas Mmp2 is required to limit ECM to the pre-luminal and luminal domain.

### CB polarization requires Mmp2 activity

Apicalisation of Integrin is an early event in CB polarization [34]. Localised Integrin enables CBs to stabilise polarising morphogens such as Slit and its receptor, Robo, which are required for lumen formation [34–37]. However, it remains unclear what signals coordinate early Integrin apicalisation. Since Vkg accumulates ectopically in *mmp2* mutants (Fig 2C–2C”, Fig 3G and 3G’), we sought to determine whether Integrin localisation could be informed by Mmp activity. In wildtype hearts, βPS Integrin localises to the luminal domain (Fig 3A and 3A’). In *mmp1* mutants, βPS is restricted to the reduced lumen (Fig 3B and 3B’), whereas in *mmp2*, and *mmp1*,*mmp2* mutants, βPS accumulates at the apical and basal sides of the CBs and some lateral locations (Fig 3C–3D’). These results suggest Mmp2 may restrict Integrin ligand binding to the luminal domain by negative regulation of ECM assembly elsewhere.

**Fig 4:**
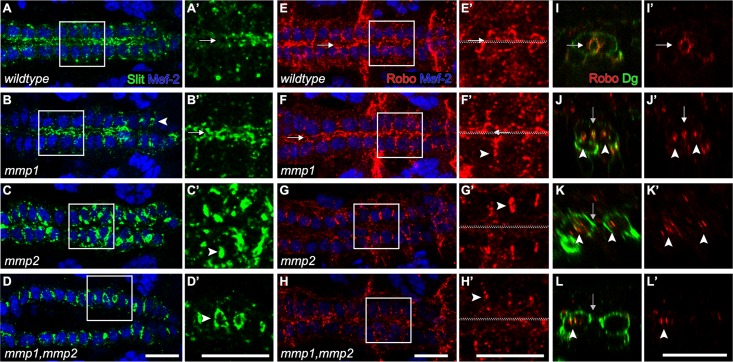
Slit and Robo localise to ectopic lumens in *mmp* mutants. (A-H) Wildtype and *mmp* mutant embryos labelled with α -Slit and α -Robo. (I-L) Cross-sectional view of wildtype and *mmp* mutant embryos labelled with α-Dg and α-Robo. (A) In wildtype, Slit localises to the luminal domain of the stage 17 CBs (arrow). (B) In *mmp1* mutants, Slit localises to the luminal domains (arrow), however lateral mislocalisation is also observed (arrowhead). (C,D) In *mmp2* and *mmp1*,*mmp2* mutants, Slit is mislocalised, accumulating ectopically at the lateral domains of the CBs (arrowheads). (E, I-I’) In wildtype, Robo localises to the luminal domain (arrows). (F,J-J’) In *mmp1* mutants, apical (arrow) and lateral (arrowhead) distribution of Robo is observed. In *mmp2* (G,K-K’) and *mmp1*,*mmp2* (H,L-L’) mutants, Robo localises to the lateral domains of CBs (arrowhead), whereas Dg is distributed throughout the plasma membrane (arrow). Scale—10 μm.

Signalling by the Slit morphogen is required for heart lumen formation, perhaps by destabilising Cadherin based CB adhesion [35–37]. If ECM assembly is altered in Mmp mutants, then the resulting expansion of Integrin distribution would extend the domain of Slit and Robo stabilisation. In wildtype stage 17 embryos, Slit and Robo accumulate along the apical domain and the lumen develops as a single, continuous midline tube (Fig 4A, 4E, 4I and 4I’). In *mmp1* mutants, Slit and Robo localise predominantly to the luminal domain, however lateral accumulation is occasionally observed (Fig 4B, 4F, 4J and 4J’). More dramatically, in *mmp2* and *mmp1*,*mmp2* mutants, Slit and Robo fail to apicalise, but accumulate at the lateral domains (Fig 4C, 4D, 4G, 4H and 4K–4L’). These lateral domains resemble ectopic pockets with luminal composition. Integrin and Vkg are present in the ectopic lumens in *mmp2* mutants, confirming the luminal characteristics of these lateral structures (data not shown). Even though Integrin localisation includes the apical domain in *mmp* mutants, the ability to direct Slit and Robo to the apical domain is lost in *mmp2* and *mmp1*,*mmp2* mutants and retained in *mmp1* mutants. Collectively these results suggest that targeting of guidance molecules Slit/Robo to the luminal domain, downstream of Integrin function, requires Mmp2 activity.

In the *Drosophila* nervous system, Mmp2 dependent cleavage of Faulty Attraction, an ECM protein containing multiple epidermal growth factor (EGF) domains, is required for motor axon targeting [19]. Similarly, Slit, an EGF domain containing protein, can be cleaved in to a C-terminus fragment of unknown function and an active N-terminus fragment [38, 39]. Since Slit normally localises to the luminal domain we sought to establish if Mmp2 processed Slit outside the lumen. However, subsequent to ubiquitous transgene expression in embryos, no differences in full-Slit or C-Slit levels in Mmp2 overexpressing embryos were seen relative to control (data not shown). If Slit processing by Mmp2 is unlikely, other mechanisms account for Slit mislocalisation in *mmp2* mutants. One possibility is that lateral accumulation of Slit and Robo in *mmp2* and *mmp1*,*mmp2* mutants is due to perturbed Integrin dependent targeting and ectopic stabilisation.

### Mmp1 and Mmp2 are required for the motility of CB Leading Edge during collective cell migration

**Fig 5:**
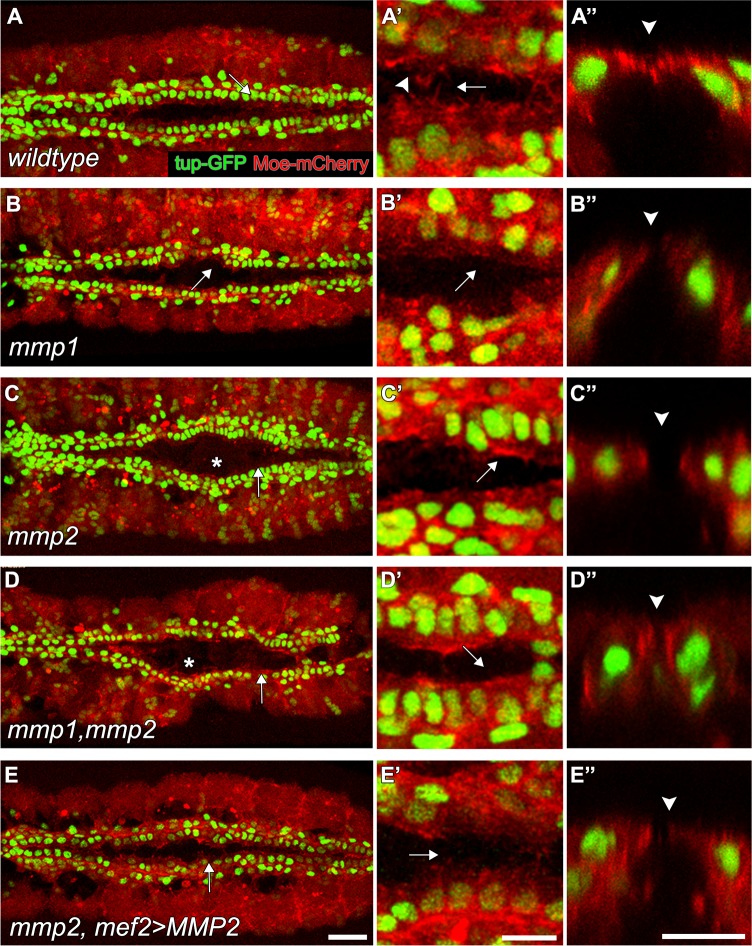
MMPs are required for the formation of filopodia and lamellopodia at the leading edge. Live embryos expressing *tup-GFP* (to visualise CB nuclei) and *UAS-Moesin-mCherry* under the control of *mef2-GAL4* driver are visualised. Thin cytoplasmic protrusions were identified as filopodia, whereas sheet-like extensions were categorised as lamellopodia. (A-A”) In wildtype embryos, CBs extend finger-like filopodia (arrow) and sheet-like lamellopodia (arrowhead) towards their contralateral partner cells. CBs make contact at the dorsal midline (arrowhead). (B-B”) In *mmp1* mutants, filopodial and lamellopodial activity at the leading edge is reduced (arrow) and CBs form short outgrowths towards their contralateral partners (arrowhead). (C-D”) In *mmp2* and *mmp1*,*mmp2* mutants delayed migration is observed (asterisk). CBs extend reduced numbers of filopodia and lamellopodia (C’,D’ arrow). CBs appear rounded without apical extensions towards the midline (C”,D” arrowheads). (E-E”) Expression of a *mmp2* transgene in the CBs of *mmp2* mutants restores formation of filopodia, lamellopodia (arrows) and apical outgrowths (arrowhead). Scale—25 μm in A-E and 10 μm in A’-E”.

**Fig 6:**
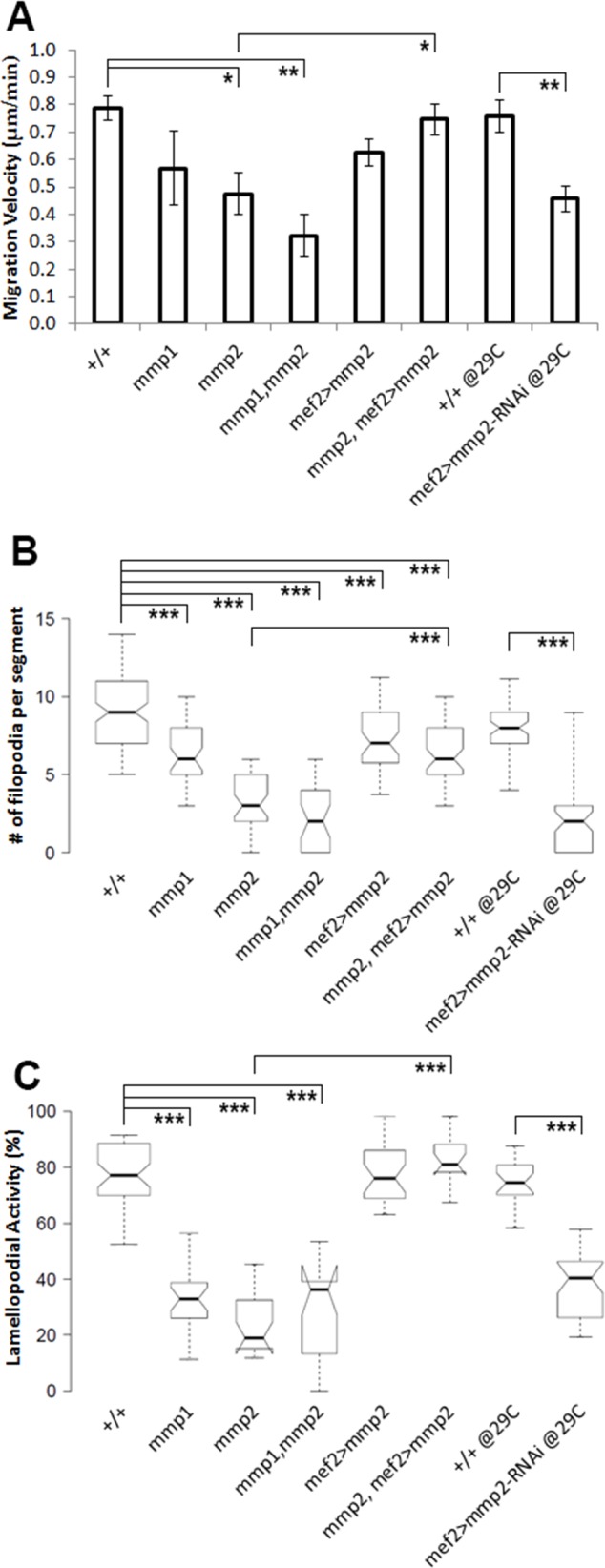
Migration velocity, filopodial and lamellopodial activity of CBs are reduced in *mmp* mutant embryos. Analysis of time-lapse movies of live embryos expressing cardiac markers *tup*-GFP (nuclear) and *mef2*>*moesin*-mCherry. (A) Migration velocity is significantly reduced in *mmp2*, *mmp1*,*mmp2* and *mmp2-RNAi* knockdown embryos. Knockdown and control embryos were raised at 29°C to maximise the effect of the GAL4/UAS system. Bars represent mean±s.e.m. Filopodial (B) and lamellopodial (C) activities are significantly reduced in *mmp1*, *mmp2*, *mmp1*,*mmp2 mutants* and *mmp2-RNAi* knockdown embryos. Migration velocity (A), filopodial (B) and lamellopodial (C) activities are restored in embryos where MMP2 rescue was performed. For Box plots, center lines show the medians and box limits indicate the 25th and 75th percentiles as determined by R software [60]. Whiskers extend to the 5^th^ and 95^th^ percentile. Width of the boxes is proportional to the square root of the sample size. Notches represent 95% confidence intervals. One-tailed T-test was performed to determine significance of difference. *—P<0.05, **—P<0.01, ***—P<0.001.

CBs collectively migrate towards their final destination—the dorsal midline. Since Mmps regulate cell migration in multiple vertebrate and invertebrate models [40–42], we assessed whether either Mmp contributes to CCM of CBs. To observe CCM, we analysed time-lapse movies of heart development in embryos expressing a nuclear marker, tail-up-GFP (tupGFP), and a fluorescent actin binding protein, Moesin-mCherry (MoeRFP) (Fig 5). This method allowed us to quantify aspects of CCM such as migration velocity, filopodial and lamellopodial activity [43]. We noticed a significant reduction in migration velocity in both *mmp2* and *mmp1*,*mmp2* mutant CBs, and a non-significant reduction was observed in *mmp1* mutants (Fig 6A, S2 Table). These results strongly suggest that ECM modification is required for the normal migration of CBs. To better understand why migration speed declined, we quantified the number of filopodia and lamellopodia at the Leading Edge (LE). As CBs approach the midline, the number of Moesin-labelled filopodia and lamellopodia increases at the LE (Fig 5A and 5A’, S2A Fig, S5 Movie). These protrusions mediate the formation of apical adhesions with the contralateral CBs upon medial contact (Fig 5A”) [36]. In *mmp1*, *mmp2*, and *mmp1*,*mmp2* mutants, filopodial and lamellopodial activities were significantly reduced (Fig 5B, 5B’,5C, 5C’, 5D and 5D’, Figs 6B and 5C, S6–S8 Movies, S2 Table). Although both Mmp1 and Mmp2 activity were required to promote LE activity and outgrowth formation during CCM stages of heart development, only Mmp2 was required for timely migration of the CBs. Additionally, the negative correlation between distance to the midline and number of filopodial extensions was reduced in *mmp1*, *mmp2* and *mmp1*,*mmp2* mutants compared to wildtype (S2B–S2D Fig, S1 Table). During early stages of migration, CBs maintain direct contact with Amnioserosa cells and follow behind the ectoderm LE as it migrates towards the dorsal midline [44]. However, following ectoderm dorsal closure, CBs initiate autonomous movement to complete their migration to the midline. We used complimentary approaches to test whether Mmp2 is autonomously required in the CBs for CCM and lumen formation. First, we attempted to rescue *mmp2* mutants by expressing a *UAS-Mmp2* transgene using a mef2-GAL4 construct that drives the expression of GAL4 in heart and body wall muscle cells, but not in the Amnioserosa or the Ectoderm which are immediately ventral and dorsal to the heart tissue. Migration velocity, filopodial, lamellopodial activity of CBs and lumen formation (data not shown) were rescued (Fig 5E–5E”, Fig 6A–6C). The negative correlation between distance to the midline and number of filopodia per segment however was not restored (S2F Fig, S1 Table). Second, we reduced Mmp2 levels in the CBs by driving a UAS-*mmp2*-RNAi construct under the control of *mef2*-GAL4 driver. Recapitulating the phenotype of *mmp2* mutants, CCM of CBs was delayed and frequent gaps (data not shown) and clumps of CBs were also observed in *MMP2* reduced embryos (S3B Fig, S9 Movie). Reduction of filopodial and lamellopodial activities were also apparent and migration velocity was reduced (Fig 6A–6C). Formation of intermittent lumens was observed in Mmp2 reduced embryos (S3F Fig). Altogether these results suggest that CBs autonomously regulate CCM and lumenogenesis primarily via Mmp2 activity.

### Overexpression of Mmp2 in the CBs disrupts ECM, CCM and lumen formation

**Fig 7:**
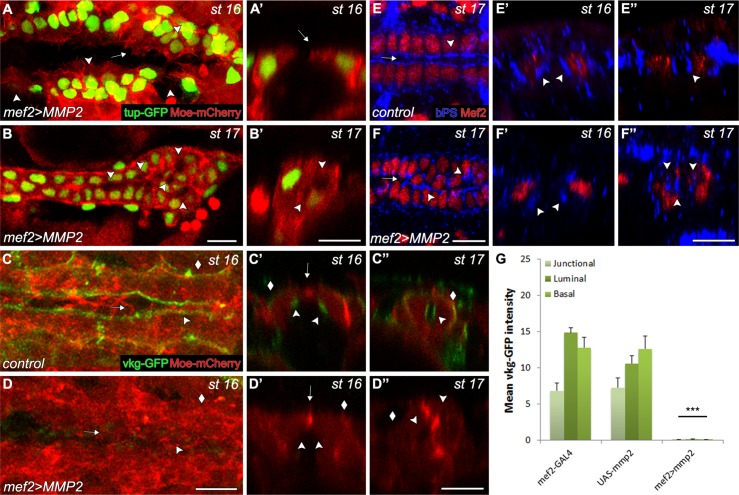
Overexpression of Mmp2 in the CBs disrupts ECM, cell migration and lumen formation. UAS-*mmp2* transgene was expressed in the CBs under the control of *mef2*-GAL4. (A-A’) Filopodial, lamellopodial activities and outgrowth formation are not affected (arrows). However, alignment of the bilateral row of nuclei is disrupted (arrowheads). (B-B’) Multiple ectopic lumens form at the heart proper region when MMP2 is overexpressed (arrowheads). (C-D”) Embryos expressing *vkg*-GFP and *mef2*>*moesin*-mCherry demonstrate the reduction of Vkg-GFP levels at the CB ECM in *mef2>mmp2* embryos relative to control (arrows). In control embryos, ECM localises at the pre-luminal (C,C’ arrowhead), luminal (C”, arrowhead) and basal domains (C-C”, diamonds). After Mmp2 overexpression, ECM is reduced at the pre-luminal (D,D’ arrowhead), luminal (D”, arrowhead) and basal domains (D-D”, diamonds) (C-D’, arrow indicates the dorsal midline). (E-F’) Embryos labelled with α-βPS and α-Mef2 reveal the disorganization of CBs at the heart proper in Mmp2 overexpressing hearts relative to control (arrows). In control embryos, Integrin localises to the pre-luminal (E’ arrowheads) and luminal domains (E,E”, arrowheads). In *mef2>mmp2* embryos, Integrin localises to the pre-luminal domain (F’, arrowheads) and ectopic lumens (F,F”, arrowhead). (G) Mean Vkg-GFP fluorescence intensity in control and Mmp2 overexpressing embryos for junctional, luminal and basal domains. Junctional, luminal and basal fluorescence intensity, respectively, differ significantly in Mmp2 overexpressing embryo compared to both controls as determined by one-tailed T test. ***—p<0.001. Scale– 10 μm.

The observation that migratory and lumen formation phenotypes in *mmp1* mutant were relatively less pronounced compared to *mmp2* mutants suggests that Mmp2 plays the predominant role in regulating cardiac development. Since removing Mmp2 activity in the CBs results in disruption of CCM, lumen formation and accumulation of ECM around the CBs, we hypothesised that elevating Mmp2 levels in the CBs would increase ECM degradation and decrease the size of the CB lumen. We employed a *UAS-Mmp2* construct under the control of heart specific *mef2*-GAL4. During CCM, the regular CB alignment was disrupted, but the disjointed CB rows were still able to extend filopodia and lamellopodia (Fig 7A and 7A’, S10 Movie). Quantification further demonstrated that migration velocity and lamellopodial activity in the CBs were not affected, however filopodial activity was reduced (Fig 6A–6C). The negative correlation between distance to the midline and number of filopodia per segment was also reduced (S2E Fig, S1 Table). Once migration was complete, the dramatic misalignment of the CB rows persisted in to late embryonic stages (Fig 7B and 7B’). We further assessed whether Integrin localisation was affected in the CBs. In control and Mmp2 overexpressing embryos, Integrin localised to the pre-luminal and luminal domains of the CBs (Fig 7E–7E” and 7F–7F”). At stage 17, in Mmp2 overexpressing embryos however, Integrin was observed at multiple small lateral and midline luminal pockets (Fig 7B and 7B’). Consistent with a role for Mmps in ECM degradation, overexpression of Mmp2 resulted in severe reduction of VkgGFP levels surrounding the CBs relative to control embryos (Fig 7C–7C” 7D–7D”, 7G). This suggests that presence of Vkg in the CB pre-luminal ECM is not essential for Integrin targeting but might be required for expansion of luminal ECM. However, disruption of the ECM during lumen formation fragments the continuous Integrin-ECM complex and generates ectopic luminal pockets.

### Ectopic expression of TIMP in the ectoderm inhibits lumen formation in CBs

**Fig 8:**
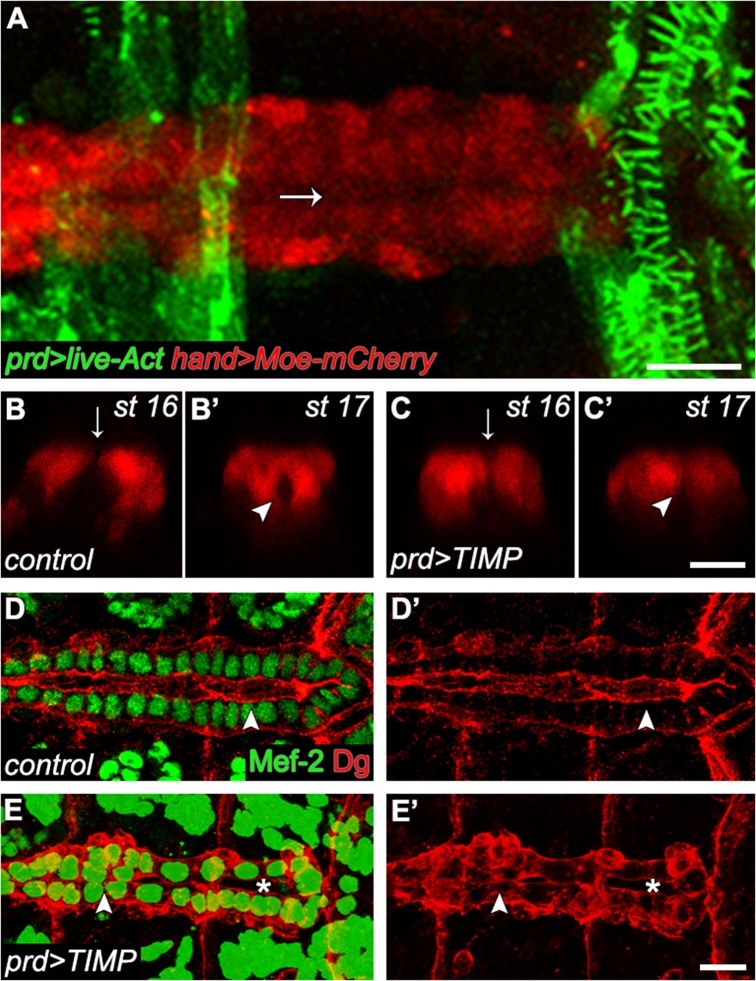
Inhibition of MMPs by ectopic expression of TIMP in the ectoderm disrupts heart lumen formation. (A) *prd-GAL4* expression is reported by *UAS-live-Actin-GFP* (green) and CBs are expressing Moesin-mCherry under the control of *hand* promoter. Arrow indicates the CB midline. (B-C’) Cross sectional images were taken through the non-ostial cells under the non-*paired* expressing ectoderm. (B-B’) In stage 16 control embryos, CBs extend protrusions (arrow) towards the contralateral partner cells and by stage 17 form an enclosed lumen (arrowhead). (C-C’) When TIMP is expressed in paired expressing ectodermal stripes, apical protrusions (arrow) do not form and lumen formation does not occur (arrowhead). (D-D’) In stage 17 control embryos, Dg localises to the apical luminal domain of CBs (arrow). (E-E’) In embryos expressing TIMP ectopically, accumulation of Dg is observed around the CBs and a medial lumen fails to form (arrow). Gaps between contralateral CBs are also observed (asterisk). Scales—10 μm.

Mmp activity is regulated by Timp which inhibits both Mmp1 and Mmp2 in *Drosophila* [10]. Timps are expressed in both mammalian and *Drosophila* hearts during cardiac development [45, 46], suggesting that CBs tightly regulate Mmp activity to control ECM assembly. Surprisingly, overexpressing Timp in the CBs using a *mef2-GAL4* driver did not affect CCM or lumen formation (data not shown). Therefore, we expressed Timp ectopically in the tissue closest to the apical extensions of the CBs where Mmp2 accumulates. We used the *paired*-GAL4 driver to express Timp to higher levels in the ectodermal stripes immediately dorsal to the heart (Fig 8A). We reasoned that since Mmp2 is detected at the apical domain of CBs, expression of TIMP in the dorsally located ectodermal stripes would be sufficient to inhibit local Mmp2 activity. All CBs, located ventral to the paired expressing and the more distant non-paired expressing ectodermal stripes, failed to extend apical protrusions and form a lumen when Timp was ectopically expressed (Fig 8B, 8B’, 8C and 8C’). Next, we assessed whether CBs retained their ability to localise ECM receptor Dg to the appropriate CB domain. In the control embryo, Dg localised to the luminal and basal domains of the CBs (Fig 8D and 8D’). Subsequent to ectopic Timp expression, CBs failed to form a lumen and Dg label was dispersed around the CBs (Fig 8E and 8E’). In addition, misaligned and rounded CBs were also observed, phenocopying *mmp2* and *mmp1*,*mmp2* mutants. This phenotype, coupled with the *in situ* hybridisation data [45], suggests that Timp modulates Mmp function during heart development, and that expression by the CBs is spatially regulated.

## Discussion

Multiple Mmps and Timps are expressed in embryonic vertebrate hearts and are required for key events such as tube formation, cardiac looping and heart septation [20, 21]. Although vertebrate studies highlight the importance of Mmps and Timps, the large number of Mmp and Timp genes complicate analysis of their function. Employing *Drosophila* as a genetic model, we demonstrate that Mmps are required for CCM, ECM degradation, cell polarization and lumen formation in *Drosophila* hearts.

Loss of Mmp2 activity results in severe embryonic heart development phenotypes that persist to larval stages. This activity is autonomously required in the CBs. Surprisingly, despite a malformed heart, *mmp2* mutant larvae appear healthy and develop through to pupation, whereas *mmp1* and *mmp1*,*mmp2* mutant larvae die during late larval stages [17]. This suggests that an effective heart is not required for embryonic and larval development. Peristaltic movements within the larva may be sufficient to circulate the hemolymph around the larval body in the absence of a functional heart.

### Mmp1 and Mmp2 have distinct roles during embryonic heart development

Previous studies suggest that Mmp1 and Mmp2 play distinct roles in *Drosophila*. During fat body disintegration in pupae, Mmp2 is required to cleave components of the basement membrane and thereby disable cell-ECM interaction while Mmp1 disrupts cell-cell adhesions by cleaving cell adhesion molecule, E-Cadherin [30]. Consistent with this, our data indicates that Mmp1 and Mmp2 perform divergent functions during heart development, specifically during CCM and lumen formation. Mmp1 is required to expand lumen size, which is consistent with E-cadherin as a key substrate [30]. Since Mmp1 localises at the pre-luminal and luminal domains, its activity may restrict E-cadherin to less exposed dorsal and ventral attachment sites and remove E-cadherin from the more exposed medial luminal domain. In a complementary manner, Mmp2 localises to the apical outgrowths in CBs and assists in clearing the Collagen-IV containing apical ECM from the LE. The significant reduction of filopodia and lamellopodia at the LE of CBs and accumulation of Vkg around the CBs in *mmp2* mutants suggests that Mmp2 activity promotes the formation of invasive protrusions by degrading the ECM barrier at the dorsal apical domain. Enlargement of the Cadherin domain in *mmp1* mutants may reduce the concentration of LE motility factors below threshold for filopoda formation.

The contrasting roles for Mmp1 and Mmp2 reflect different substrate specificities and localization during heart development. Mmp1 can degrade Collagen-IV and Fibronectin *in vitro*, however cannot degrade laminins, Fibrinogen or type I and type II fibrillar collagens [8]. Mmp2 on the other hand is proposed to degrade Collagen-IV as well as laminins [9]. Laminins are required for proper localisation of ECM components around the CBs and are required for heart development [27, 28, 47, 48]. Stability of Laminin is key to lumen formation. In both *lanb1* and *mmp2* mutants, abnormal accumulation of Collagen-IV is observed around the CBs [47].

**Fig 9:**
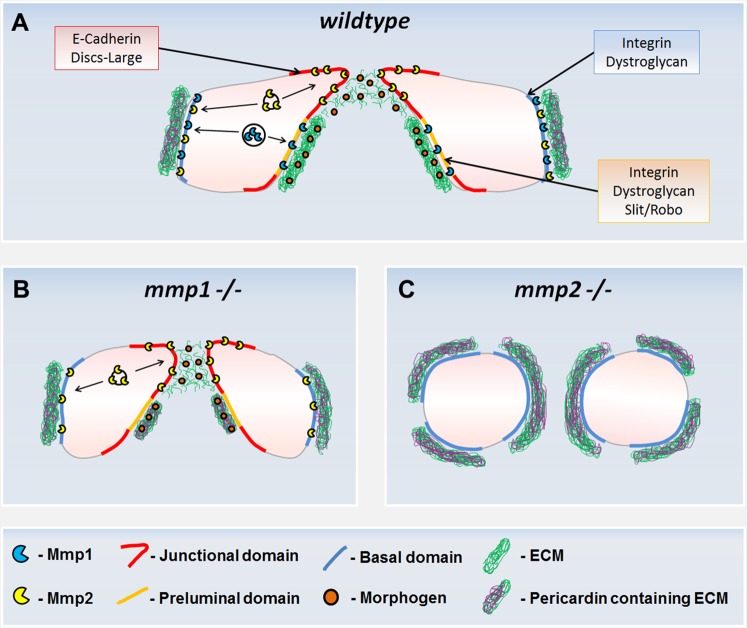
A model describing the mechanism of Mmp1 and Mmp2 function during CCM of CBs. (A) In wildtype stage 16 embryos visualised in cross-section, Mmp2 is targeted to the LE where it cleaves components of the ECM, allowing for stabilisation of a Cadherin domain, and releasing embedded morphogens, possibly Slit, which trigger filopodia and lamellopodia formation. Mmp1 is targeted to the preluminal domain, where it excludes assembly of Cadherin-based adherens junctions and partakes in homeostatic ECM turnover. Pericardin localization is limited to the basal side of the CBs. (B) In *mmp1* loss-of-function embryos, Pericardin is present at a reduced luminal ECM, suggesting Mmp1 negatively regulates the size of the Cadherin domain and Pericardin deposition. Mmp2 is likely targeted to the LE where it degrades ECM components and releases embedded morphogens. (C) In *mmp2* loss of function embryos, the apical identity of both junctional and preluminal domain is disrupted suggesting Mmp2 is essential for Integrin and Slit mediated CB polarization.

In mammals, GPI anchored MT1-Mmps accumulate at podosomes of normal cells and invadopodia of metastasising cancer cells and are responsible for removing the basement membrane barrier [13, 14, 49]. CBs extend apical outgrowths, morphologically similar to mammalian podosomes, which localise Mmp2. Therefore, Mmp2 may weaken or remove the apical ECM barrier to create the necessary environment for the formation of actin-based membrane extensions (Fig 9). Degradation of the ECM might further release embedded signalling molecules such as Slit and Netrin which are required for formation of filopodia and lamellopodia in the CBs [43].

### How might Mmps promote cell polarization?

An early feature of CCM of CBs is the targeting of Integrin and ECM components to the apical pre-luminal domain [34]. Subsequently, Integrin at the pre-luminal domain stabilises lumen size determinants, Slit and Robo. In wildtype heart tubes, the apical domain is comprised of the luminal sub-domain bracketed by dorsal and ventral junctional domains that seal the heart tube medially. In *mmp1* mutants this fundamental polarity is maintained, but the luminal sub-domain is reduced and the junctional domains are extended. In contrast, in *mmp2* mutants, apical junctional domains are not established and a modified ECM domain extends over the entire CB apical surface. The *mmp2* mutant apical ECM contains ectopic Pericardin, and Slit and Robo do not accumulate; neither is characteristic of a pre-luminal domain. Mmp2 is not required to target Integrin apically, since βPS1 accumulates apically in mutants. Rather, Mmp2 is required to limit the extent of the ECM within this apical domain. Mmp2 is also not required for proper targeting of Integrin in the *Drosophila* pupal fat body [30]. Both Mmp function and Integrin accumulation at the incipient CB apical surface are required to stabilise (and possibly target) the luminal determinants Robo and Slit [34]. In both the fat body and heart, Integrin localisation is not affected when Mmp2 is overexpressed suggesting that Mmp2 activity regulates Slit/Robo apicalisation downstream of Integrin activity and that Integrin is not a target substrate for Mmp2 in *Drosophila*. The spatial restriction of Fibroblast Growth Factor signalling activity by Mmps described during branching morphogenesis in *Drosophila* [50] may analogously apply to heart lumen formation signals Netrin and Slit.

Mmp2 sculpts the location and molecular composition of the apical CB lumen. In *mmp2* mutant embryos, ectopic lumens (described by [51, 52]) form between lateral CBs which localise luminal markers, Integrin, Dg, Slit, Robo and Vkg. This suggests that the complex of luminal determinants can assemble in the absence of Mmp2, and thereby displace Cadherin from lateral pockets. In converse, increased Mmp2 expression results in degradation of Collagen-IV on all cell surface domains. Collagen-IV is not required for formation of luminal space between CBs since lateral luminal pockets containing Integrin and not Collagen-IV are also observed between Mmp2 overexpressing CBs.

Ectopic lumens, containing the canonical luminal determinants Slit and Robo, form when Mmp2 is absent or if Mmp2 is overexpressed. Both perturbations alter the molecular identity of the apical surface, but this does not reveal how pre-luminal Integrin leads to Slit and Robo accumulation. Two possible mechanisms emerge. The Syndecan family of proteoglycans work cooperatively with Integrins in ECM adhesion [53]. Syndecans are substrates of MT1-Mmps [41, 54] and stabilise Slit and Robo in the heart lumen [55]. Therefore, it is possible that *Drosophila* Sdc is an Mmp2 substrate, and its cleavage would prevent Slit accumulation. Alternatively, Timp may be localised by a component of the pre-luminal ECM and acts locally to inhibit Mmp2 activity.

## Conclusion

Heart morphogenesis in vertebrates and *Drosophila* begins with polarised cell migration, medial cardiac cell fusion and lumen formation. Mmp action plays a key role in all three activities. Mmps regulate the sub-cellular pattern of ECM stabilisation and the local molecular composition of the ECM. This is required to localise membrane responses to guidance signals, including those for LE motility and for lumen specification.

## Methods and materials

### *Drosophila melanogaster* strains

*mmp1*^*2*^, *mmp1*^*Q112*^, *mmp2*^*DfUba1*^, *mmp2*^*w307*^, *UAS-TIMP*, *UAS-mmp2 and UAS-mmp2-RNAi* (NIG-1794-1R-1) lines were obtained from A. Page-McCaw [17–19]. *Tail-up-F4-GFP* was provided by R. Schulz [26]. *UAS-moesin-mCherry* was provided by T. Millard [56]. *paired*-GAL4 was provided by B. Reed [57]. Remaining stocks were obtained through the Bloomington stock center (NIH P40OD018537).

### Immunohistochemistry

Embryo and larval fixation and staining were adapted from standard protocols [58]. The primary antibodies used were rabbit α-Mef2 (1:5,000 [34]), rabbit α-Dg (1:150,[51]), chicken α-GFP (1:1,000, (Novus Biologics)), α-Mmp1 (1:10, 1:1 cocktail of 3A6B4 and 3B8D12, Drosophila Studies Hybridoma Bank (DSHB)), α-Prc (EC11),α-Robo (13C9), α-Dlg (4F3), α-Slit (C555.6D) and α-βPS (CF.6G11) (all 1:30, DSHB). Alexa 488, 546, 594, and 647 secondary antibodies were used at a 1:150 dilution (Molecular Probes). Images were acquired using a Leica SP5 confocal microscope. Transverse images are single optical sections, while all other frontal images are projections of four to six sections. All images were processed using ImageJ and assembled with Adobe Photoshop.

### Time-lapse live imaging, quantification and statistics

Embryos were prepared according to the hanging drop protocol [59]. Mutants were identified by the absence of GFP expressing balancer. Live imaging was performed using a Leica SP5 confocal microscope.

Quantification of Viking levels was performed by measuring the GFP intensity of a 2 μm^2^ area at the junctional, luminal or basal domains of CBs by employing ImageJ. All values were normalised to wildtype embryos (*vkgGFP/+*) (n = 19 embryos).

Quantification of filopodial and lamellipodia activity is as previously described by Raza and Jacobs [43]. One-tailed T-tests were performed to compare migration velocity, filopodial and lamellopodial activity between a specific pair of genotypes, with a p threshold of 0.05. Single cross-sectional images at the heart proper region per embryos were scored blindly and the averaged values are shown in Table 1.

## Supporting information

S1 Fig Mmp1 localization at the dorsal midline during embryogenesis. Dorsal (A-A’,C-C’) and cross sectional (B-B’,D-D’) view of embryos immunolabelled with α-Mmp1 and α-Dg antibodies are shown. Mmp1 localizes to the pre-luminal (A-A’,B-B’, arrow) and basal (A-A’,B-B’, arrowhead) domain during migratory stages. At stage 17, Mmp1 localizes to the luminal domain of CBs (C-C’,D-D’,arrows). Scale– 10 μm
(TIF)

S2 Fig Scatter plot of ‘distance of the cardiac segment to the midline’ for each segment and the ‘number of filopodia extended in cardiac segments’. In wildtype embryos (A), an inverse correlation is observed between distan. The inverse correlation between ‘distance to the midline’ and the ‘number of filopodia of the segment’ is lost in *mmp1* (B), *mmp2* (C), *mmp1*,*mmp2* (D) and *mef2>mmp2* (E) embryos. Expression of Mmp2 transgenes under *mef2* control in *mmp2* mutants (F) partially restores the inverse correlation between ‘distance to the midline’ and ‘number of filopodia per segment’ relative to respective mutants.
(TIF)

S3 Fig *MMP2* RNA depletion in the CBs disrupts filopodial and lamellopodial activities and lumen formation. *UAS-mmp2-RNAi* was expressed under the control of *mef2-GAL4* driver. LE of CBs extends multiple filopodia and lamellopodia (A arrow). In MMP2 depleted embryos, filopodial and lamellopodial activity of the CBs is reduced (B arrow). (C-D) CBs do not extend protrusions towards contralateral partners when MMP2 is downregulated (D arrow) compared to control (C arrow). (E-F) Lumen formation does not occur in embryos where MMP2 is downregulated (F arrowhead) compared to control (E arrowhead). Accumulation of actin at the junctional domain is reduced in embryos where MMP2 is downregulated (E,F arrow). Scale– 10 μm.
(TIF)

S1 Movie Cardiac ECM in wildtype embryo. Time-lapse of embryos expressing Vkg-GFP construct. Vkg localises to the pre-luminal domain at the apical, and basal side of the CBs. Embryonic hemocytes migrate underneath the heart. All time-lapse movies were filmed over 30 minutes. A z-stack of 20–30 μm with 1 μm intervals was acquired every minute and Z projected. Movies are 3 fps or a time compression of 180 fold. Posterior of the heart is to the right in this and subsequent movies. Scale—25 μm for all movies.
(MP4)

S2 Movie Cardiac ECM in *mmp1* mutant embryo. Vkg-GFP is present at the apical and basal side of the CBs, however migration appears disrupted and delayed.
(MP4)

S3 Movie Cardiac ECM in *mmp2* mutant embryo. Apical accumulation of Vkg-GFP is observed. The polarised positioning of Vkg-GFP around the CBs is affected.
(MP4)

S4 Movie Cardiac ECM in *mmp1,mmp2* mutant embryo. The ECM around the CBs is severely affected. Migration of the CBs is delayed and disrupted.
(MP4)

S5 Movie CCM of CBs in wildtype embryo. Time-lapse of embryos expressing a nuclear marker, *tup*-GFP and an actin binding fluorescent marker, *moesin*-mCherry. CBs extend multiple filopodial and lamellopodial extensions at the LE and migrate towards the dorsal midline while maintaining adhesion with ipsilateral cells.
(MP4)

S6 Movie CCM of CBs in *mmp1* mutant embryo. Filopodial and lamellopodial activity of the CB LE is significantly reduced, however, some filopodial and lamellopodial processes are observed. A gap spanning 8 cell diameter is observed in the bottom LE.
(MP4)

S7 Movie CCM of CBs in *mmp2* mutant embryo. Filopodial and lamellopodial activity of the CB LE is significantly reduced, however, some filopodial and lamellopodial processes are observed. Migration of the CBs is delayed.
(MP4)

S8 Movie CCM of CBs in *mmp1,mmp2* mutant embryo. Filopodial and lamellopodial activity of the CB LE is significantly reduced, however, some filopodial and lamellopodial processes are observed. Migration of the CBs is delayed. The bilateral row structure of the heart is disrupted and clumping of CBs is noted.
(MP4)

S9 Movie CCM of CBs in embryos expressing UAS-*MMP2*-RNAi under the control of *mef2*-GAL4. Filopodial and lamellopodial activity of the CB LE is significantly reduced, however, some filopodial and lamellopodial processes are observed. Migration of the CBs is delayed. The bilateral row structure of the heart is disrupted and clumping of CBs is noted.
(MP4)

S10 Movie CCM of CBs in embryos expressing UAS-*MMP2* under the control of *mef2*-GAL4. Filopodial and lamellopodial protrusions are observed at the CB LE. The bilateral row structure of the heart is disrupted and clumping of CBs is noted.
(MP4)

S1 Table Comparison of correlation coefficients of ‘distance of CBs to the midline’ and ‘number of filopodia per heart segment’ in wildtype, mutant and rescued embryos. Z values were obtained through Fischer r to z transformation. Z values were used to determine the significance of difference (p-value). ‘N’ represents the number of heart segments scored.
(DOCX)

S2 Table Sample size for migration velocity, filopodial activity and lamellopodial activity quantification. Genotypes which display a significant difference in correlation coefficient compared to wildtype are shaded (p<0.05, -1.645<z<1.645).
(DOCX)
