# Supplementary figures and images for: Matrix Metalloproteinases are required for membrane motility and lumenogenesis during Drosophila heart development
Source: PLoS One. 2017 Feb 13;12(2):e0171905. doi: 10.1371/journal.pone.0171905 (PMC5305246; doi:10.1371/journal.pone.0171905)

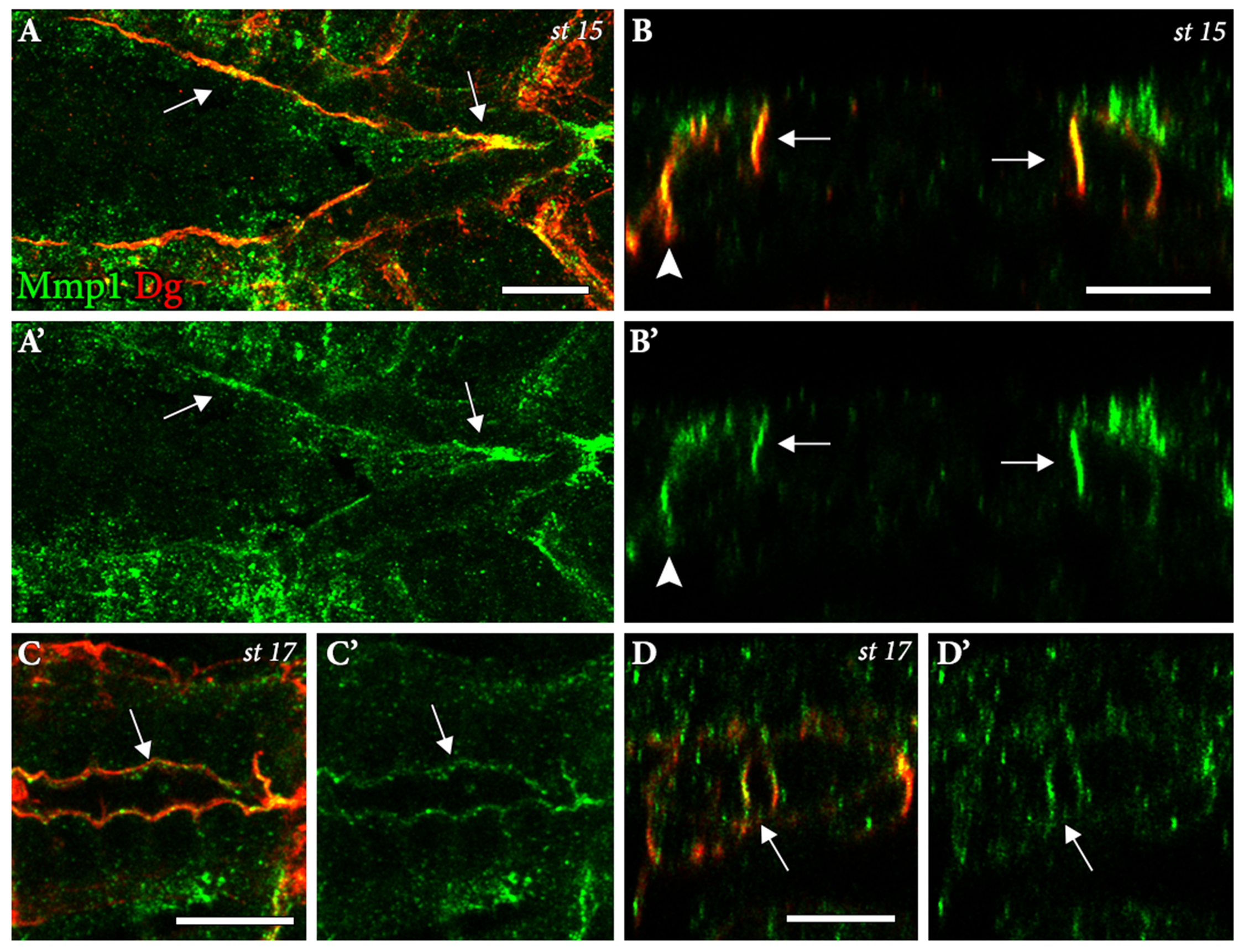

Supplement: S1 Fig — Dorsal (A-A’,C-C’) and cross sectional (B-B’,D-D’) view of embryos immunolabelled with α-Mmp1 and α-Dg antibodies are shown. Mmp1 localizes to the pre-luminal (A-A’,B-B’, arrow) and basal (A-A’,B-B’, arrowhead) domain during migratory stages. At stage 17, Mmp1 localizes to the luminal domain of CBs (C-C’,D-D’,arrows). Scale– 10 μm (TIF) [file pone.0171905.s001.tif]

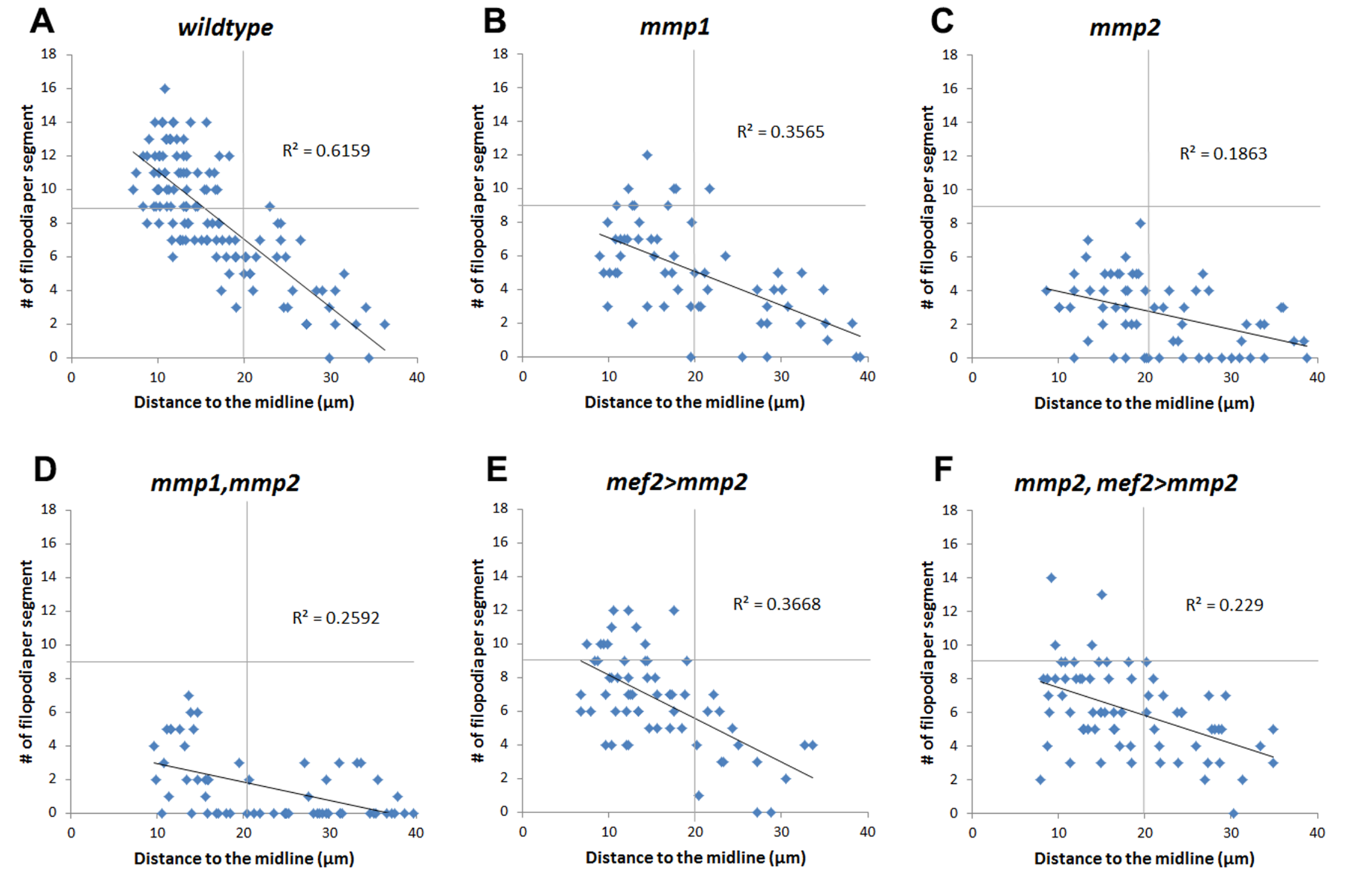

Supplement: S2 Fig — In wildtype embryos (A), an inverse correlation is observed between distan. The inverse correlation between ‘distance to the midline’ and the ‘number of filopodia of the segment’ is lost in mmp1 (B), mmp2 (C), mmp1,mmp2 (D) and mef2>mmp2 (E) embryos. Expression of Mmp2 transgenes under mef2 control in mmp2 mutants (F) partially restores the inverse correlation between ‘distance to the midline’ and ‘number of filopodia per segment’ relative to respective mutants. (TIF) [file pone.0171905.s002.tif]

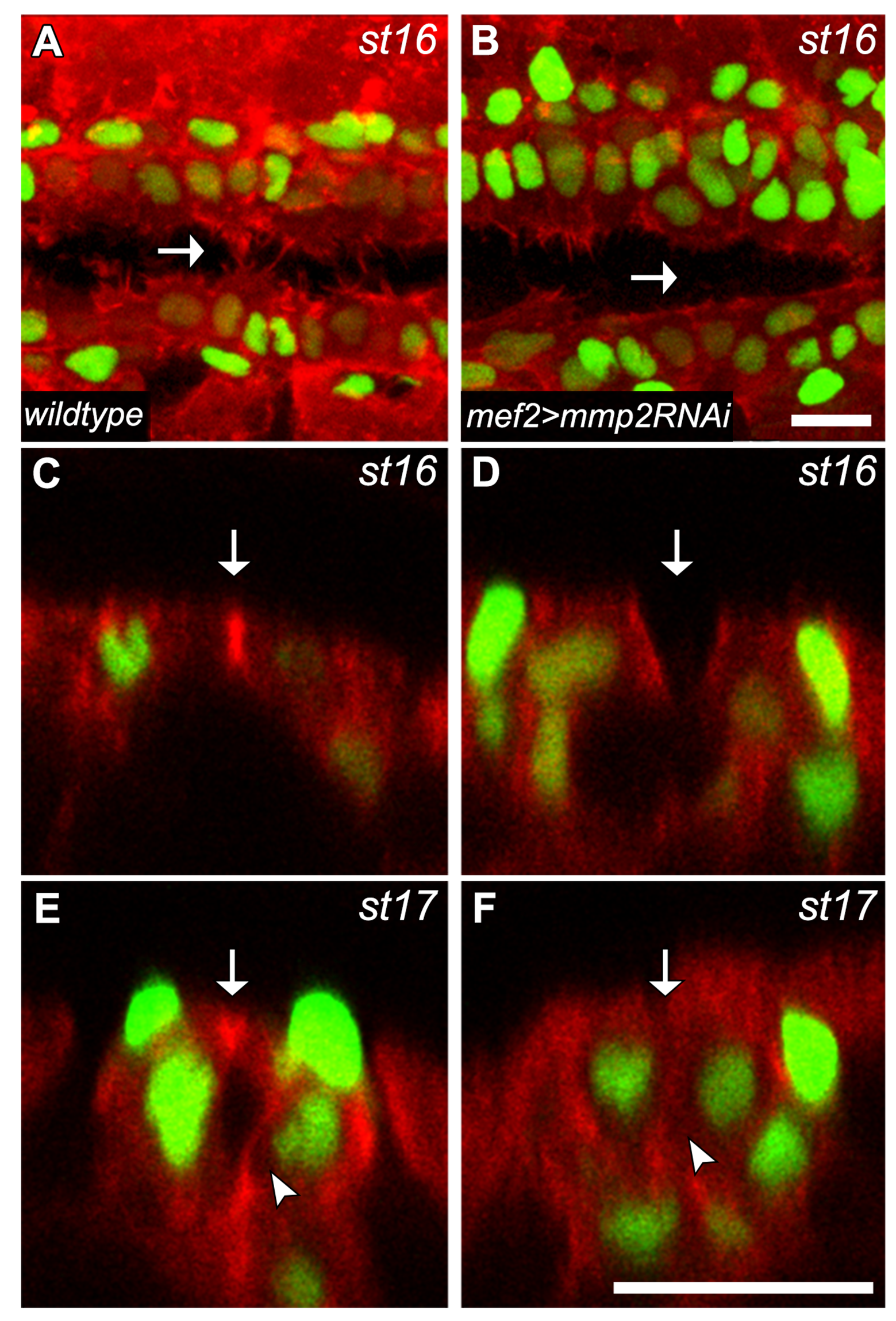

Supplement: S3 Fig — UAS-mmp2-RNAi was expressed under the control of mef2-GAL4 driver. LE of CBs extends multiple filopodia and lamellopodia (A arrow). In MMP2 depleted embryos, filopodial and lamellopodial activity of the CBs is reduced (B arrow). (C-D) CBs do not extend protrusions towards contralateral partners when MMP2 is downregulated (D arrow) compared to control (C arrow). (E-F) Lumen formation does not occur in embryos where MMP2 is downregulated (F arrowhead) compared to control (E arrowhead). Accumulation of actin at the junctional domain is reduced in embryos where MMP2 is downregulated (E,F arrow). Scale– 10 μm. (TIF) [file pone.0171905.s003.tif]
