# Supplementary material for: Matrix Metalloproteinases are required for membrane motility and lumenogenesis during Drosophila heart development
Source: PLoS One. 2017 Feb 13;12(2):e0171905. doi: 10.1371/journal.pone.0171905 (PMC5305246; doi:10.1371/journal.pone.0171905)
Supplement: S1 Table — Z values were obtained through Fischer r to z transformation. Z values were used to determine the significance of difference (p-value). ‘N’ represents the number of heart segments scored. (DOCX) [file pone.0171905.s014.docx]

**S1 Table**. Comparison of correlation coefficients of ‘distance of CBs to the midline’ and ‘number of filopodia per heart segment’ in wildtype, mutant and rescued embryos. Z values were obtained through Fischer r to z transformation. Z values were used to determine the significance of difference (p-value). Cells containing significant p-values are shaded in grey. ‘N’ represents the number of heart segments scored.

| ***Genotypes*** | **Embryos scored for Migration Velocity** | **Segments scored for Filopodial Act.**  **(# of embryos)** | **LEs scored for Lamellopodial Activity**  **(# of embryos)** |
| --- | --- | --- | --- |
| *wildtype* | 16 | 110 (16) | 48 (8) |
| *mmp1* | 7 | 42 (7) | 24 (4) |
| *mmp2* | 6 | 45 (6) | 24 (4) |
| *mmp1,mmp2* | 6 | 35 (6) | 22 (4) |
| *mef2>mmp2* | 5 | 56 (5) | 30 (5) |
| *mmp2,mef2>mmp2* | 7 | 54 (7) | 18 (3) |
| *+/+ @29C* | 5 | 58 (5) | 24 (4) |
| *mef2>mmp2RNAi @29C* | 10 | 57 (10) | 36 (6) |

* - z and p values are relative to wildtype

◊ - z and p values are relative to respective mutants
