# Supplementary material for: Matrix Metalloproteinases are required for membrane motility and lumenogenesis during Drosophila heart development
Source: PLoS One. 2017 Feb 13;12(2):e0171905. doi: 10.1371/journal.pone.0171905 (PMC5305246; doi:10.1371/journal.pone.0171905)
Supplement: S2 Table — Genotypes which display a significant difference in correlation coefficient compared to wildtype are shaded (p<0.05, -1.645<z<1.645). (DOCX) [file pone.0171905.s015.docx]

**S2 Table. Sample size for migration velocity, filopodial activity and lamellopodial activity quantification.** Genotypes which display a significant difference in correlation coefficient compared to wildtype are shaded (p<0.05, -1.645<z<1.645)

| ***Genotypes*** | **Correlation Coefficient (R)** | **N** | **z value** | **p value** |
| --- | --- | --- | --- | --- |
| *wildtype* | -0.78 | 127 | - | - |
| *mmp1* | -0.60 | 60 | -2.2^*^ | 0.01^*^ |
| *mmp2* | -0.43 | 64 | -3.74^*^ | 0.0001^*^ |
| *mmp1,mmp2* | -0.52 | 63 | -2.98^*^ | 0.001^*^ |
| *mef2>mmp2* | -0.61 | 62 | -2.13^*^ | 0.02^*^ |
| *mmp2,mef2>mmp2* | -0.48 | 70 | 0.36^◊^ | 0.36^◊^ |

* - z and p values are relative to wildtype

◊ - z and p values are relative to respective mutants
